# Self-administered acupressure training for depression in community-dwelling individuals: a randomized controlled trial and cost-effectiveness analysis

**DOI:** 10.1016/j.eclinm.2026.104023

**Published:** 2026-06-16

**Authors:** Wing Fai Yeung, Branda Yee Man Yu, Fiona Yan-Yee Ho, Carlos King Ho Wong, Ka Fai Chung, Jia Yin Ruan, Shucheng Chen, Denise Shuk Ting Cheung, Zhang-Jin Zhang, Suzanna Zick, Huilin Cheng, Yuen Shan Ho, Lai Ming Ho, Lixing Lao

**Affiliations:** a School of Nursing, The Hong Kong Polytechnic University, Hong Kong, China; b Research Centre of Chinese Medicine Innovations, The Hong Kong Polytechnic University, Hong Kong, China; c Department of Social Work and Social Administration, The University of Hong Kong, Hong Kong, China; d Department of Psychology, The Chinese University of Hong Kong, Hong Kong, China; e School of Public Health, Li Ka Shing Faculty of Medicine, The University of Hong Kong, Hong Kong, China; f Diabetes Research Centre, University of Leicester, Leicester, UK; g Department of Psychiatry, The University of Hong Kong, Hong Kong, China; h Rory Meyers College of Nursing, New York University, New York, NY, USA; i School of Nursing, Li Ka Shing Faculty of Medicine, The University of Hong Kong, Hong Kong, China; j School of Chinese Medicine, The University of Hong Kong, Hong Kong, China; k University of Michigan, Department of Family Medicine and Nutritional Sciences, Ann Arbor, MI, USA; l Virginia University of Integrative Medicine, USA

**Keywords:** Self-administered acupressure, Depression, Cost-effectiveness, Community-based intervention, Randomized control trial, Acupuncture

## Abstract

**Background:**

Depression is a detrimental mental disorder. Self-administered acupressure (SAA), a flexible self-help intervention, may be beneficial for relieving depression.

**Methods:**

A randomized controlled trial with cost-effectiveness analysis was conducted in a community setting in Hong Kong, China, between November 20, 2022, and August 19, 2024. Adults with moderate depression were randomized into SAA or mental health education (MHE) group. Participants in the SAA group underwent an acupressure training program (two 2-h sessions); they were told to practice SAA daily for 12 weeks. Participants in the MHE group received mental health education with the same schedule and duration. The primary outcome was the Patient Health Questionnaire-9 (PHQ-9) score over 12 weeks. This study was registered in ClinicalTrials.gov (#NCT05631184).

**Findings:**

A total of 250 participants (186 female [74.4%]; mean [SD] age, 41.86 [12.85] years; mean [SD] depression duration, 5.58 [6.86] years) were recruited. The SAA group (n = 125) showed a significantly greater reduction from baseline in the PHQ-9 score than the MHE group across all assessment time points (week 4: mean difference −1.04 [95% CI: −2.02 to −0.06], *d* = 0.26, P = 0.04; week 8: −1.25 [−2.39 to −0.11], *d* = 0.27, P = 0.03; week 12: −1.46 [−2.65 to −0.27], *d* = 0.31, P = 0.02). No serious adverse events were reported, although the adverse events may not have been adequately collected at all time points.

**Interpretation:**

Despite the clinically small effect sizes, the SAA training program is a potentially effective intervention for moderate depression.

**Funding:**

Health Medical Research Fund.

**Research in context**

**Evidence before this study**

Using the following two search strategies without language restrictions, we searched PubMed for studies, including clinical trials, randomized controlled trials, systematic reviews, and meta-analyses, from database inception to Jan 6, 2026: (1) (depression[tiab] OR Major depressive disorder[tiab] OR MDD[tiab]) AND (acupressure[tiab]) (both in Title/Abstract). Although previous randomized control trials have examined the effectiveness of self-administered acupressure (SAA) in different populations, including caregivers, university students, and individuals with insomnia and/or chronic conditions, none of them targeted adults with moderate depression and included health economics evaluation.

**Added value of this study**

This study is the first randomized controlled trial to evaluate the clinical and economic outcomes of an SAA training program in a community setting for adults with depression. In comparison with mental health education (MHE), the SAA intervention yielded modest but statistically significant improvements in depressive symptoms, insomnia, stress, and quality of life, enabling more participants to achieve subclinical levels of depression.

**Implications of all the available evidence**

SAA offers a feasible and potentially effective non-pharmacological option for managing moderate depression in community settings. Although the benefits of MHE are modest, SAA may serve as an accessible adjunct or early intervention strategy, particularly when stigma and barriers to conventional care persist. Future studies can explore hybrid models pairing SAA with minimal therapist guidance to optimize engagement and efficacy.

## Introduction

Depression is characterized by persistent low mood, loss of interest, sleep or appetite disturbances, and recurrent suicidal thoughts.^1^ Globally, 5.7% of adults experience depression, which is a leading cause of disability worldwide.^2^^,^^3^ In Hong Kong, the 2010–2013 Mental Morbidity Survey reported a 1-week prevalence of 2.9% for depressive episodes,^4^ whereas a recent 10-year prospective cohort study suggested that the rates of probable depression increased from 6.5% in 2017 to 11.2% in 2019.^5^ Effective conventional treatments for depression, including medication and psychotherapy, are available.^6^ However, treatments for depression are often hindered by stigma^7^^,^^8^ and potential adverse reactions.^9^ Such barriers may further prevent individuals with depression from seeking treatment^10^ or lead to discontinuation of their treatment,^11^ thereby highlighting the need for accessible, stigma-free interventions with fewer adverse reactions.

Acupressure is a non-invasive variant of acupuncture, wherein the practitioner stimulates the patient's acupoints using fingers, hands, or elbows to produce therapeutic effects. Acupressure may be a potential alternative treatment for depression. The mechanisms through which acupressure alleviates depression include neurophysiological, biochemical, and psychological processes.^12^ Acupressure, by stimulating specific acupoints, activates nerve endings and modulates the central nervous system; these effects influence the secretion of neurotransmitters such as serotonin and dopamine, while reducing the levels of stress hormones such as cortisol.^12^ Additionally, acupressure produces soothing physical sensations that contribute to psychological comfort and reduce the symptoms of depression.^13^ A meta-analysis of 19 randomized controlled trials (RCTs) indicated that practitioner-administered acupressure can reduce depressive symptoms.^14^ However, this form of practitioner-administered acupressure can be costly; therefore, it has been modified into a low-cost, self-administered approach. Previous RCTs of self-administered acupressure (SAA) involving college students,^15^ patients with multiple sclerosis,^16^ stressed caregivers,^17^ and individuals with insomnia^18^ suggest that it can improve depressive symptoms in comparison with waitlisted participants or controls receiving mental health education (MHE). However, these studies did not primarily target individuals with depression; therefore, they did not clarify the effects of SAA on depression. Therefore, we performed this RCT to examine the effectiveness of SAA delivered through a training course in relieving depression and conducted health economics analyses. We hypothesized that the SAA group would show greater improvement in depressive symptoms, as measured by the Patient Health Questionnaire-9 (the primary outcome measure), than the mental health education (MHE) group over the 12-week intervention period. The other related outcome, including researcher-rated depression severity, insomnia, anxiety, stress, and quality of life, would be compared as a secondary outcome.

## Methods

### Design overview

This assessor-blind, parallel-design RCT was reported in accordance with the Consolidated Standards of Reporting Trials (CONSORT) guidelines. Recruitment posters were placed at universities, non-governmental organizations, and on social media. After obtaining approval from the Institutional Review Board of the Hong Kong Polytechnic University (HSEARS20211213001). Written informed consent was provided from all participants before enrollment. The participants were enrolled between November 20, 2022, and August 19, 2024. The trial was registered with ClinicalTrials.gov (NCT05631184) November 20, 2022. The full trial protocol is provided in Supplementary S1.

### Participants

Eligible participants were aged between 18 and 65 years and were able to comprehend written Chinese. They were required to have a moderate level of depression, as indicated by a Patient Health Questionnaire-9 (PHQ-9) score of ≥10 (sensitivity: 0.86; specificity: 0.94)^19^ and <20 (indicating severe depression). They also had to be willing to provide informed consent and comply with the trial protocol. Exclusion criteria were as follows: recent initiation of or changes in antidepressant medication or dosage within the past three months; a previous or current diagnosis of schizophrenia, other psychotic disorders, or bipolar disorder as determined by the Chinese version of the Structured Clinical Interview for Diagnostic and Statistical Manual of Mental Disorders, 4th edition (DSM-IV), which is the most current version available in Chinese, and any suspected cases would be referred to our clinical team for a detailed assessment; a score below 22 on the Hong Kong Montreal Cognitive Assessment, suggesting cognitive impairment; the presence of skin lesions or infections at acupressure points (acupoints); significant suicidal risk as rated by a score of ≥3 on the suicide item of the Hamilton Depression Rating Scale (HDRS); pregnancy or childbearing potential without adequate contraception; and any major medical condition causing depression. The full eligibility criteria are described in Supplementary S1.

### Randomization and blinding

Participants were randomly allocated to an SAA or MHE group in a 1:1 ratio by using computer-generated block randomization with random block sizes of 4–6. Allocation concealment was maintained by using sequentially numbered, opaque, and sealed envelopes. The random sequence was generated by an independent researcher who was not involved in participant recruitment, enrollment, or assessment. The training course instructors of both groups and the research assistants responsible for telephone follow-up assessments were not blinded to the group allocation. The research personnel who performed the assessment and analysis were blinded to group allocation.

### Interventions

#### SAA

Participants attended two 2-h sessions (total, 4 h) of SAA training one week apart. The sessions were held in small groups (4–7 participants). The SAA intervention protocol was based on the traditional Chinese medicine (TCM) meridian theory and was refined by experienced acupuncturists (WFY, ZJZ). The acupoints included GV20, EX-HN3, PC6, HT7, CV6, CV4, UB23, and KI1. The acupressure technique included circular kneading or pointing (firm pressing) at these acupoints. Details of the SAA intervention protocol are provided in Supplementary S1. The selected acupoints have been commonly used for depression according to a recent systematic review^20^ and are recommended by other clinical practice guidelines.^21^^,^^22^ According to TCM theory, these acupoints collectively move stagnant *Liver-qi*, calm the *Shen* (Spirit), and tonify the *Kidney-qi* (the body's constitutional reserve). Modern neurobiological evidence suggests that needle stimulation of these points for depression, particularly GV20 and EX-HN3, may alleviate depressive symptoms by modulating the hypothalamic-pituitary-adrenal (HPA) axis and increasing the expression of brain-derived neurotrophic factor (BDNF).^23^ Additionally, points like HT7 and PC6 are associated with the regulation of neurotransmitters such as serotonin and GABA,^24^^,^^25^ as well as the stabilization of the autonomic nervous system.^26^ During the session, a handout with a step-by-step guide to SAA, including pictures showing acupoint locations and the acupressure technique, was provided to each participant. The participants practiced acupressure under the instructor's supervision, and their accuracy in performing SAA was assessed using a competency checklist. The participants were instructed to perform SAA daily for 12 weeks. During the initial week, participants also received two follow-up calls to encourage the practice and answer questions.

#### MHE

The treatment duration and frequency and the telephone follow-up schedule for the MHE group were the same as those for the SAA group (2 sessions, 2 h each). The MHE sessions were also conducted in small groups (4–7 participants). The MHE content was adapted from government public health resources^27^^,^^28^ regarding depression and mental health, which covered basic facts about mental health and depression, self-assessment, and practical strategies for maintaining well-being. Participants’ understanding was assessed through written quizzes. The training content was reviewed by a clinical psychologist (YH) and did not include other therapeutic elements, such as massage or cognitive behavioral change techniques. Details of the MHE programs are provided in Supplementary S1.

#### Fidelity

The SAA instructors were registered Chinese medicine practitioners with at least five years of clinical experience, whereas the MHE instructors were registered nurses trained by our team psychiatrist and clinical psychologist. The training materials used in both groups had been reviewed by five individuals with moderate depression (a PHQ-9 score of between 10 and 20) before trial commencement. Based on their feedback, we revised our teaching materials to replace some wording with simpler language and to provide larger, clearer diagrams of acupoint locations. To ensure consistent delivery, the principal investigator (WFY) randomly visited the sessions at least 1 per month, comprising 25% of the total 96 training sessions. A fidelity checklist was provided for both instructors and inspectors (PI) to ensure adherence to the protocol (Supplementary S2). The instructors’ role was limited to teaching the course content according to the fidelity checklist during the two initial sessions to the participants. The participants were provided with a logbook to maintain a daily record of their acupressure practice in the SAA group or their compliance with the MHE instructions in the MHE group throughout the 12-week period. Furthermore, participants in both groups received two follow-up calls from the instructors during the first week to encourage their respective practices and answer any questions.

#### Concomitant treatment

Any new pharmacological or psychological treatment for depression or changes in antidepressant dosage were documented during the assessment time points, and these participants were withdrawn from the study and the remaining assessments.

### Outcomes

Outcome measures were assessed at baseline and at weeks 4, 8, and 12 with 2-day window periods; data that were not collected within the window period were considered missing. The primary outcome was the PHQ-9 score.^29^ The PHQ-9 is a nine-item self-rated questionnaire for depression assessment. It rates depressive symptoms over the last 2 weeks on a scale of 0 (not at all) to 3 (nearly every day), with the total score ranging from 0 to 27.

The secondary outcomes included researcher-rated depression determined using the HDRS^30^; self-rated depression, anxiety, and stress determined using the Depression Anxiety Stress Scale-21 (DASS-21)^31^^,^^32^; insomnia severity based on the Insomnia Severity Index (ISI)^33^^,^^34^; and quality of life based on the Short Form Six Dimensions (SF-6D) Health Survey (SF-6D).^35^^,^^36^ These outcomes were collected by our research assistants and associates who were blinded to allocation during the assessment timepoints. Adverse events were collected during the second session and at the final follow-up assessment using an open-ended question posed by the instructors or another research assistant responsible for collecting the logbook data.

Participants’ attendance at the training course was recorded. After completion of the course, participants were asked about their willingness to learn more about self-acupressure or mental health education according to their group allocation using a question scored on a 10-point Likert scale.

### Sample size

The sample size was estimated on the basis of our previous RCT of SAA for stress.^17^ An effect size of 0.40 was used to calculate the sample size. A sample size of 125 participants per group provided 80% power to reject the null hypothesis with a significance level of 0.05, assuming a 20% dropout rate. This sample size calculation did not account for clustering within training cohorts or instructors. As a result, the effective sample size in the intervention arm may be reduced, and the study may be underpowered after accounting for intra-class correlation.

### Statistical analysis

#### Data management and analysis

The primary analysis employed an intention-to-treat approach. All statistical analyses were performed using SPSS 28.0 by a researcher blinded to group allocation through the use of ciphered code. The formal statistical analysis plan was finalized on April 29, 2024, prior to the completion of data collection and unblinding (See statistical analysis plan in Supplementary S3). Differences in the PHQ-9 scores (primary outcome) and other secondary outcomes were compared using a linear mixed-effects model (LMM) considering repeated measures and dropout and by including all available data points. Group and time points were considered as fixed factors, while participants constituted a random factor. To account for the partially nested design, an additional random effect for cohort (training group) was included in the intervention arm to model within-group clustering. This approach allows for appropriate estimation of standard errors in the presence of clustering only in the intervention arm. A significant time-by-group interaction would support our hypothesis regarding the effects of SAA. In the LMM, missing data were handled under the missing at random (MAR) assumption by including all available data points. No adjustment was made for the instructor (therapist) effect. Sensitivity analyses were performed to assess the influence of missing values on the treatment effect using multiple imputation with 10 sets of imputations based on age, sex, current antidepressant use, current medical condition, baseline PHQ-9 and HDRS scores. The interventions was further assessed by determining the proportion of participants achieving a PHQ-9 score below 5 (depression severity below mild) and compared using χ^2^ tests.^37^

#### Cost-effectiveness analysis

Quality-adjusted life years (QALYs) at baseline and follow-up assessments were calculated using the SF-6D utility scores with the area under the curve approach.^36^ The SF-6D utility values were converted into a single utility index using the Hong Kong Chinese population-specific preference-based weighting algorithm.^36^ This index ranges from 0 (death) to 1 (perfect health). The costs of implementation were assessed from the perspective of health service providers. For staff cost, the research associate documented the staff's time spent on the relevant activities for both the intervention and control groups. Then the staff's time was multiplied by the corresponding salary rate to estimate the total staff cost for each recruitment cohort. Additionally, the research associate recorded the cost of materials (e.g., printing, preparation of materials) and venue expenses based on actual spending for both groups. Incremental costs and incremental effects were compared between the SAA and MHE groups. The incremental cost-effectiveness ratio (ICER) was calculated as follows: Δ costs/Δ effects, where Δ costs represents the difference in costs between the two groups, and Δ effects represents the difference in QALYs between them. To evaluate the robustness of the ICER and quantify the uncertainty in these ratios, we conducted bootstrapping with 5000 iterations. The results were plotted in a cost-effectiveness plane. A cost-effectiveness acceptability curve displaying the probability that the intervention was cost-effective for a range of willingness-to-pay ceilings was estimated using base-case data.

### Role of the funding source

The funders had no role in the study design or in the collection, analysis, interpretation of data, writing of the report, or decision to submit the article for publication.

## Results

### Participant characteristics

**Fig. 1:**
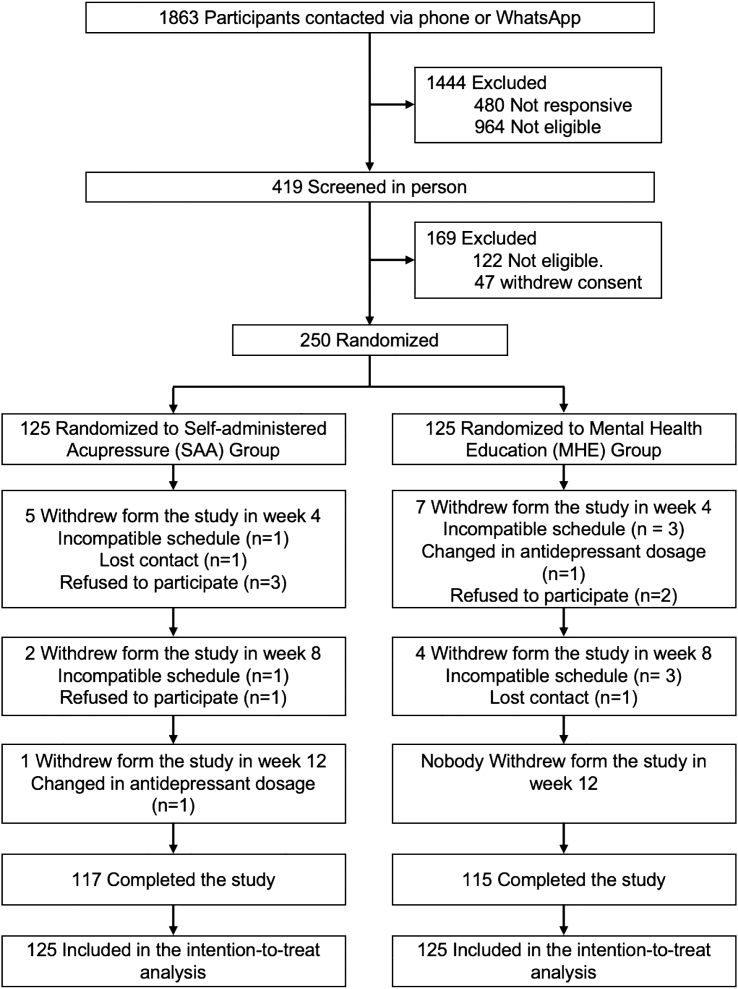
CONSORT flow diagram.

A total of 250 participants (186 female [74.4%]; mean [SD] age, 41.86 [12.85] years; mean [SD] depression duration, 5.58 [6.86] years) were randomized into the SAA or MHE group (n = 125 each) (Table 1). Nine (7.2%) and twelve (9.6%) participants withdrew from the SAA and MHE groups, respectively; the number of participants who withdrew did not differ significantly between the groups (Chi-square test, P = 0.50). The study flow diagram is shown in Fig. 1.

**Table 1:** Sociodemographic and clinical characteristics and clinical measures of the participants at baseline.

Characteristics	Total (*n* = 250)	SAA (*n* = 125)	MHE (*n* = 125)
Age (years), mean (SD)	41.86 (12.85)	41.42 (12.79)	42.30 (12.94)
Sex, *n* (%)			
Female	186 (74.4)	92 (73.6)	94 (75.2)
Education level, *n* (%)			
Secondary or below	64 (25.6)	29 (23.2)	35 (28.0)
Tertiary or above	186 (74.4)	96 (76.8)	90 (72.0)
Marital status, *n* (%)			
Single/Divorced/Widowed	153 (61.2)	78 (62.4)	75 (60.0)
Married	97 (38.8)	47 (37.6)	50 (40.0)
Occupation, *n* (%)			
Employed	148 (59.2)	74 (59.2)	74 (59.2)
Freelancer	3 (1.2)	2 (1.6)	1 (0.8)
Unemployed	18 (7.2)	11 (8.8)	7 (5.6)
Homemaker/Student/Retired	81 (32.4)	38 (30.4)	43 (34.4)
Current psychiatric disorders, *n* (%)^a^	63 (25.2)	24 (19.2)	39 (31.2)
Current antidepressant use, *n* (%)	53 (21.2)	21 (16.8)	32 (25.6)
Current medical conditions, *n* (%)	62 (24.8)	35 (28.0)	27 (21.6)
Outcome measures			
PHQ-9 score, mean (SD)	13.36 (2.56)	13.36 (2.57)	13.35 (2.55)
ISI score, mean (SD)	15.55 (5.21)	15.10 (5.38)	16.00 (5.00)
DASS-21			
Depression subscore, mean (SD)	19.30 (8.25)	19.95 (8.34)	18.64 (8.14)
Anxiety subscore, mean (SD)	16.20 (8.10)	15.73 (7.75)	16.67 (8.45)
Stress subscore, mean (SD)	24.94 (7.76)	25.38 (7.29)	24.51 (8.22)
SF-6D score, mean (SD)	0.67 (0.07)	0.67 (0.07)	0.67 (0.07)
HDRS score, mean (SD)	13.22 (4.44)	13.38 (4.58)	13.06 (4.31)

Abbreviations: DASS-21, Depression Anxiety Stress Scale-21; HDRS, Hamilton Depression Rating Scale; ISI, Insomnia Severity Index; MHE, mental health education; PHQ-9, Patient Health Questionnaire-9; SAA, self-administered acupressure; SD, standard deviation; SF-6D, Short Form 6 Dimensions.

a All psychiatric disorders diagnosed were major depressive disorders.

### Treatment compliance

A total of 118 (94.4%) and 114 (91.2%) participants completed the two training sessions in the SAA group and MHE group, respectively. The ratings on degree of willingness to attend similar training courses in the SAA and MHE groups were 9.29 (SD = 1.25) and 8.30 (SD = 1.54), respectively. In the SAA group, 77 participants (61.6%) performed acupressure at least 4 days per week during the 12-week period. The average duration of self-practice at home was 20.83 (SD = 8.86) min per day. The compliance in each assessment time point can be found in sTable S1 in Supplementary S4.

### Effectiveness

#### Primary outcome

The differences in PHQ-9 scores between the SAA and MHE groups across the assessment time points were compared using the LMM (Table 2). The analysis included all randomized participants. The SAA group showed significantly greater reductions than the MHE group in the PHQ-9 score across all the assessment time points, and the between-group difference in changes from baseline was −1.04 at week 4 (n = 120 in SAA; n = 118 in MHE: 95% CI: −2.02 to −0.06; *d* = 0.26, P = 0.04), −1.25 at week 8 (n = 118 in SAA; n = 114 in MHE: 95% CI: −2.39 to −0.11; *d* = 0.27, P = 0.03), and −1.46 at week 12 (n = 117 in SAA; n = 115 in MHE: 95% CI: −2.65 to −0.27; *d* = 0.31, P = 0.02; Table 2). The results of the primary analysis based on the original statistical analysis plan are presented in sTable S2. Sensitivity analyses using multiple imputations for missing values demonstrated that these between-group differences showed a similar magnitude. Additional sensitive analysis was done by incorporating baseline psychiatric disorder and antidepressant use as covariates in the LMM. The intervention effect remained statistically significant, suggesting that the potential baseline imbalance did not substantially influence the primary results (sTable S3 in Supplementary S4). The SAA group showed a PHQ-9 score reduction of −5.47 points from baseline to week 12, which met the minimal clinically important difference threshold of 5 points.^38^

**Table 2:** Depression outcomes across the assessment time points.

	SAA group (n = 125) Mean (SE)^a^	SAA group change from the baseline Mean (SE)	MHE group (n = 125) Mean (SE)^a^	MHE group change from the baseline Mean (SE)	Between-group differences in changes from the baseline (95% CI)	Effect size (d)^b^	P value^c^
PHQ-9 score^d^							
Baseline	13.36 (0.36)	–	13.35 (0.36)	–	–	–	0.07^l^
Week 4^e^	9.44 (0.37)	−3.92 (0.35)	10.47 (0.37)	−2.88 (0.35)	−1.04 (−2.02, −0.06)	0.26	0.04
Week 8^f^	8.89 (0.37)	−4.47 (0.41)	10.13 (0.38)	−3.22 (0.41)	−1.25 (−2.39, −0.11)	0.27	0.03
Week 12^g^	7.89 (0.37)	−5.47 (0.43)	9.34 (0.38)	−4.01 (0.43)	−1.46 (−2.65, −0.27)	0.31	0.02
HDRS score^h^							
Baseline	13.38 (0.71)	–	13.06 (1.03)	–	–	–	0.02^l^
Week 4^i^	8.94 (0.71)	−4.15 (0.40)	10.56 (1.04)	−2.50 (0.41)	−1.65 (−2.78, −0.52)	0.36	0.004
Week 8^j^	8.85 (0.71)	−4.23 (0.46)	10.35 (1.04)	−2.70 (0.46)	−1.53 (−2.80, −0.25)	0.30	0.02
Week 12^k^	7.95 (0.71)	−5.14 (0.47)	9.62 (1.04)	−3.44 (0.48)	−1.70 (−3.02, −0.38)	0.32	0.01

Abbreviations: CI, confidence interval; HDRS, Hamilton Depression Rating Scale; MHE, mental health education; PHQ-9, Patient Health Questionnaire-9; SAA, self-administered acupressure; SE, standard error.

a Estimated mean and standard error from the linear mixed-effects model.

b Effect size (ES) based on the mean change from the baseline in the treatment group minus the mean change from baseline in the control group, divided by the pooled standard deviation of score change. The ES was categorized as small (ES = 0.2), medium (ES = 0.5), and large (ES = 0.8; Cohen, 1988). A positive sign indicates a highly favorable effect in the intervention group.

c The P value for the interaction between groups and assessment time points in the linear mixed-effects model.

d The Instructor level variance = 0.00; Instructor intra-class correlation = 0.00.

e Due to missing, n = 120 in the SAA group; n = 118 in the MHE group.

f Due to missing, n = 118 in the SAA group; n = 114 in the MHE group.

g Due to missing, n = 117 in the SAA group; n = 115 in the MHE group.

h The Instructor level variance = 0.90; Instructor intra-class correlation = 0.04.

i Due to missing, n = 120 in the SAA group; n = 116 in the MHE group.

j Due to missing, n = 118 in the SAA group; n = 112 in the MHE group.

k Due to missing, n = 117 in the SAA group; n = 112 in the MHE group.

l The P value for the interaction between groups and all assessment time points in the linear mixed-effects model.

Within the SAA group, exploratory analysis indicated a trend toward greater PHQ-9 reductions among participants practicing ≥4 days per week compared with those practicing <4 days per week (−6.12 vs. −4.44; independent t-test, P = 0.050). Daily practice duration of SAA was weakly but significantly correlated with PHQ-9 score reduction (r = −0.183, P = 0.045), whereas the frequency of practice (total number of days with practice) was not significantly correlated (r = −0.176, P = 0.050).

#### Secondary outcomes

In comparison with the MHE group (Table 3), the SAA group showed a significantly greater improvement in the researcher-rated HDRS score at week 4 (n = 120 in SAA; n = 116 in MHE: −1.65; 95% CI: −2.78 to −0.52; *d* = 0.36, P = 0.004), week 8 (n = 118 in SAA; n = 112 in MHE: −1.53; 95% CI: −2.80 to −0.25; *d* = 0.30, P = 0.02), and week 12 (n = 117 in SAA; n = 112 in MHE: −1.70; 95% CI: −3.02 to −0.38; *d* = 0.32, P = 0.01). The intervention also showed superior effects on reducing the DASS-depression subscore (sample size ranged from 117 to 120 in SAA, and from 111 to 116 in MHE; *d* = 0.27−0.35, all P < 0.05) and DASS-stress subscore (sample size ranged from 117 to 120 in SAA, and from 111 to 116 in MHE; *d* = 0.29–0.42, all P < 0.05) at all assessment time points. Although the DASS-anxiety subscores showed improvement in both groups, the between-group differences were not statistically significant (P = 0.16). The SAA group showed a significantly greater reduction in the ISI score than the MHE group, with between-group differences ranging from −1.36 (n = 120 in SAA; n = 115 in MHE: P = 0.02) at week 4, −1.86 (n = 118 in SAA; n = 112 in MHE: P = 0.005) at week 8, and −1.80 (P = 0.008) at week 12 (n = 117 in SAA; n = 112 in MHE: *d* = 0.30 – 0.38). The SAA group showed a more significant enhancement in quality of life than the MHE group, as measured by the SF-6D score at week 4 (n = 121 in SAA; n = 116 in MHE: *d* = 0.30, P = 0.02), and week 12 (n = 117 in SAA; n = 111 in MHE: *d* = 0.39, P = 0.002). However, the enhancement in the quality of life by the SAA group was non-significant at week 8 (*d* = 0.23, P = 0.07). The results of the primary analysis based on the original statistical analysis plan are presented in sTable S4.

**Table 3:** Other study outcomes across the assessment time points.

	SAA group (n = 125) Mean (SE)^a^	SAA group change from the baseline Mean (SE)	MHE group (n = 125) Mean (SE)^a^	MHE group change from the baseline Mean (SE)	Between-group differences in changes from the baseline (95% CI)	Effect size (d)^b^	P value^c^
ISI^d^							
Baseline	15.10 (0.50)	–	16.00 (0.50)	–	–	–	0.02^n^
Week 4^e^	10.95 (0.50)	−4.15 (0.40)	13.21 (0.51)	−2.79 (0.41)	−1.36 (−2.48, −0.24)	0.30	0.02
Week 8^f^	10.43 (0.50)	−4.67 (0.46)	13.19 (0.51)	−2.81 (0.47)	−1.86 (−3.14, −0.57)	0.38	0.005
Week 12^g^	9.37 (0.51)	−5.73 (0.48)	12.07 (0.51)	−3.93 (0.48)	−1.80 (−3.14, −0.47)	0.34	0.008
DASS-21 Anxiety subscore^d^							
Baseline	15.73 (0.71)	–	16.67 (0.71)	–	–	–	0.57^n^
Week 4^h^	11.79 (0.72)	−3.94 (0.55)	13.12 (0.72)	−3.56 (0.56)	−0.38 (−1.92, 1.16)	0.06	0.63
Week 8^i^	10.69 (0.72)	−5.04 (0.67)	12.54 (0.73)	−4.13 (0.68)	−0.91 (−2.78, 0.96)	0.12	0.34
Week 12^j^	9.48 (0.73)	−6.25 (0.71)	11.85 (0.74)	−4.82 (0.73)	−1.43 (−3.44, 0.58)	0.18	0.16
DASS-21 Depression subscore^d^							
Baseline	19.95 (0.77)	–	18.64 (0.77)	–	–	–	0.02^n^
Week 4^h^	13.87 (0.78)	−6.08 (0.62)	14.98 (0.78)	−3.67 (0.63)	−2.42 (−4.14, −0.69)	0.35	0.006
Week 8^i^	13.49 (0.78)	−6.46 (0.75)	14.49 (0.80)	−4.15 (0.77)	−2.31 (−4.42, −0.20)	0.27	0.03
Week 12^j^	11.33 (0.79)	−8.63 (0.81)	13.18 (0.80)	−5.47 (0.82)	−3.16 (−5.43, −0.89)	0.35	0.007
DASS-21 Stress subscore^d^							
Baseline	25.38 (0.78)	–	24.51 (0.78)	–	–	–	0.007^n^
Week 4^h^	19.94 (0.79)	−5.44 (0.65)	21.46 (0.80)	−3.05 (0.66)	−2.38 (−4.21, −0.55)	0.34	0.01
Week 8^i^	19.53 (0.80)	−5.84 (0.77)	20.92 (0.81)	−3.59 (0.79)	−2.25 (−4.42, −0.09)	0.29	0.04
Week 12^j^	16.68 (0.80)	−8.70 (0.81)	19.52 (0.82)	−4.99 (0.83)	−3.71 (−5.99, −1.42)	0.42	0.002
SF-6D score^d^							
Baseline	0.67 (0.015)	–	0.67 (0.021)	–	–	–	0.01^n^
Week 4^k^	0.66 (0.015)	−0.015 (0.009)	0.62 (0.021)	−0.045 (0.009)	0.029 (0.005, 0.054)	0.30	0.02
Week 8^l^	0.68 (0.015)	0.010 (0.010)	0.65 (0.021)	−0.018 (0.011)	0.027 (−0.002, 0.056)	0.23	0.07
Week 12^m^	0.71 (0.015)	0.036 (0.010)	0.65 (0.021)	−0.012 (0.011)	0.048 (0.017, 0.078)	0.39	0.002

Abbreviations: CI, confidence interval; DASS-21, Depression Anxiety Stress Scale-21; ISI, Insomnia Severity Index; MHE, mental health education; SAA, self-administered acupressure; SE, standard error; SF-6D, Short Form 6 Dimensions.

a Estimated mean and standard error from the linear mixed-effects model.

b Effect size (ES) based on the mean change from the baseline in the treatment group minus the mean change from baseline in the control group, divided by the pooled standard deviation of score change. The ES was categorized as small (ES = 0.2), medium (ES = 0.5), or large (ES = 0.8; Cohen, 1988). A positive sign indicates a highly favorable effect in the intervention group.

c The P value for the interaction between groups and assessment time points in the linear mixed-effects model.

d The Instructor level variance = 0.00; Instructor intra-class correlation = 0.00.

e Due to missing, n = 120 in the SAA group; n = 115 in the MHE group.

f Due to missing, n = 118 in the SAA group; n = 112 in the MHE group.

g Due to missing, n = 117 in the SAA group; n = 112 in the MHE group.

h Due to missing, n = 120 in the SAA group; n = 116 in the MHE group.

i Due to missing, n = 118 in the SAA group; n = 112 in the MHE group.

j Due to missing, n = 117 in the SAA group; n = 111 in the MHE group.

k Due to missing, n = 121 in the SAA group; n = 116 in the MHE group.

l Due to missing, n = 118 in the SAA group; n = 112 in the MHE group.

m Due to missing, n = 117 in the SAA group; n = 111 in the MHE group.

n The P value for the interaction between groups and all assessment time points in the linear mixed-effects model.

The proportion of participants in the SAA group who achieved a PHQ-9 score of <5 at week 12 was significantly higher than that in the MHE group (24.8% vs 13.6%, P = 0.03; sTable S5 in Supplementary S4).

### Adverse events

Adverse events (AEs) were collected during the second session and at the final follow-up assessment. Seventeen (13.6%) participants in the SAA group reported AEs, including pain at the acupoints (n = 6), finger joint pain (n = 4), abdominal distension after pressing (n = 3), dizziness after pressing (n = 2), tinnitus during pressing (n = 1), and constipation (n = 1). Except for constipation, the aforementioned AEs were considered at least probably related to the SAA intervention. One participant in the MHE group reported feeling drowsy, likely due to abstaining from coffee. All the reported AEs were mild in severity and resolved without medical intervention. None of them led to dropout. However, the AEs were assessed using open-ended questions, which may not adequately capture all relevant information. Therefore, records of such AEs need to be interpreted with caution.

### Cost-effectiveness analysis

In this 12-week study, the SAA group had a mean QALY of 0.6733 (SD = 0.0924), which was 0.0301 higher than that of the MHE group (mean = 0.6433, SD = 0.0868). The average cost of the SAA intervention was HK$1105 (SD = HK$237), which was higher than the cost for the MHE group (mean = HK$997; SD = HK$217). Additionally, the ICER for the SAA in comparison with MHE was HK$3599.4 per QALY gained.^39^

**Fig. 2:**
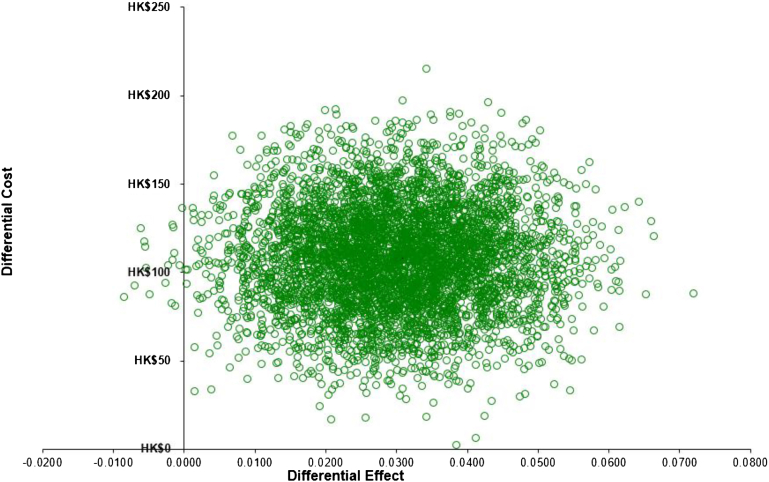
Cost-effectiveness plane.

This value falls significantly below the willingness-to-pay threshold of US$17,409–28,801 in Hong Kong.^40^ A sensitivity analysis was conducted using 5000 bootstrapping iterations to evaluate the cost-effectiveness ratio between SAA and MHE. The resulting cost-effectiveness plane (Fig. 2) indicated that in comparison with MHE, SAA yielded greater gains in QALY and had higher costs, since most of the cost–effect pairs were in the upper right quadrant. The parametric cost-effectiveness analysis curve based on the base-case data showed that the probability of SAA being deemed cost-effective was 80% at a willingness-to-pay threshold of HK$6000 (US$769) per QALY gained (Supplementary Figure S1). As the willingness-to-pay threshold increased to HK$8000 per QALY gained (US$1026, approximately 2% of HK GDP per capita),^39^ the probability of SAA being cost-effective increased to over 90%.

## Discussion

This RCT demonstrated that the SAA training program is a feasible non-pharmacological intervention for community-dwelling adults with moderate depression. Throughout the 12-week period, the SAA training program yielded greater, but modest, improvements than the MHE program in relieving depressive symptoms, as measured by both self-rated and blinded researcher-rated scales. The between-group difference in the change in the PHQ-9 score at week 12 was statistically significant but small (−1.46 points; Cohen's d = 0.30), indicating a modest incremental benefit of SAA over MHE. The SAA group showed a reduction of −5.47 in the PHQ-9 score at week 12, meeting the minimal clinically important difference threshold of 5 points. Moreover, a higher proportion of participants in the SAA group achieved a subclinical level of depression. In addition, participants in the SAA group showed significantly better improvements in insomnia and stress, and had a better quality of life than those in the MHE group. Given the small between-group effect size for the PHQ-9 score, these findings should be interpreted as evidence of modest incremental benefit rather than a large standalone effect. Although the SAA training program yielded more QALYs than the MHE program, it incurred higher costs than the later; however these economic findings should be interpreted with caution, given the small absolute QALY gain and the context-specific cost assumptions.

To our knowledge, this study is the first RCT with health economics analyses to evaluate the clinical outcomes and economic implications of an SAA training program for depression. In comparison with MHE, the SAA intervention demonstrated a small effect size in reducing depressive symptoms, as evidenced by self-reported PHQ-9 scores (*d* = 0.27–0.30) and blinded researcher-rated HDRS scores (*d* = 0.30–0.36) at each assessment time point. These findings align with the results of a recent meta-analysis, which supported the efficacy of practitioner-delivered acupressure as an adjunct to conventional treatments.^14^ The meta-analysis reported a substantially large effect size in terms of the reductions in HDRS scores when practitioner-administered acupressure was combined with conventional care (standardized mean difference [SMD] = 1.23 vs. conventional treatment alone; SMD = 0.95 vs. emotional care). The discrepancy in effect sizes between our study and previous studies may be due to methodological differences. Earlier trials employed practitioner-delivered acupressure as an adjunct treatment to conventional treatments (e.g., medications); however, our trial examined acupressure as a self-guided intervention. Self-administered modalities often yield smaller effects because of challenges in adherence, technique accuracy, and the absence of clinician monitoring, which are critical to behavioral interventions.^41^ Nevertheless, even modest effect sizes hold public health significance for scalable, low-cost interventions such as SAA, particularly in resource-limited settings where access to professional care is constrained.^42^ In this context, SAA can be viewed as providing a modest additional benefit over MHE rather than as a replacement for standard treatments. Future studies can explore hybrid models pairing SAA with minimal therapist guidance to optimize engagement and efficacy, as seen in digitally delivered cognitive behavioral therapies.^43^

A critical challenge in evaluating the efficacy of acupressure is distinguishing specific therapeutic effects from non-specific factors, such as practitioner–participant interactions. The use of sham acupressure, although proposed as an alternative,^44^ remains contentious in acupuncture-related studies because of the complex nature of the intervention.^45^ The inherent characteristics of sham acupressure, such as acupoint palpation or physical pressure, may elicit non-specific physiological responses, thereby blurring the line between “active” and “control”.^46^, ^47^, ^48^ Furthermore, the self-administered nature of acupressure complicates blinding because participants can verify intervention details easily (e.g., via online resources), resulting in unsuccessful blinding. Previous trials employing care-as-usual or waitlisted controls failed to account for these non-specific effects, potentially overestimating the intervention effect. For instance, the use of waitlisted controls creates nocebo effects by depriving participants of perceived therapeutic engagement, whereas care-as-usual fails to standardize the interaction time.^49^ To address these limitations, our study employed MHE as a control to match the instructor-contact time and session structure in the SAA group. This design controlled the non-specific effects (e.g., attention and social support) arising from practitioner engagement and minimized the nocebo risks associated with untreated control groups by equating the interaction duration and format. This design also enabled control of contextual factors, which is essential for robustly attributing observed outcomes to the SAA intervention.

The MHE group showed improvements in depressive symptoms and other secondary outcomes, thereby narrowing the between-group differences. This observation aligns with previous studies where education-based comparison groups showed similar reductions in symptoms.^50^ Although such improvements can partially reflect the natural course of depression, regression to the mean, or non-specific therapeutic factors (e.g., attention and group support), the MHE content itself may play an active role. MHE typically includes education on depression etiology, symptom recognition, and lifestyle modifications (e.g., diet, physical activity, and sleep hygiene),^51^ which may empower individuals to adopt self-management strategies and reduce maladaptive coping behaviors. These lifestyle modifications may help reduce depressive symptoms.^52^, ^53^, ^54^ Thus, MHE can be further examined as an accessible and acceptable early intervention for community-dwelling individuals with moderate depression.

Our exploratory analysis suggests a correlation between SAA compliance and improvement in PHQ-9 scores. The frequency of practice and time spent on SAA were potentially associated with a greater intervention effect, suggesting that consistent adherence to the SAA practice enhances its therapeutic effects. A previous qualitative research identified barriers to SAA compliance, such as the burden of daily logbook entries, scheduling difficulties, time constraints, participants' inertia, and the perceived complexity of acupressure techniques.^55^ Future studies should incorporate reminders via instant messaging apps,^56^ telehealth coaching sessions to provide ongoing technical support,^57^ or a simplified point-selection protocol to mitigate these barriers, thereby enhancing participant compliance and optimizing the efficacy of AA.

Our findings demonstrated that SAA is a feasible non-pharmacological intervention for depression. Acupressure usually is not associated with the adverse effects commonly observed in acupuncture treatment, such as bleeding and bruising at the acupoint site.^58^^,^^59^ The most common adverse events reported in this study, finger joint pain and pain at acupoints, were likely caused by improper technique or prolonged pressure on acupoints, which have also been reported in previous trials.^60^, ^61^, ^62^ A small proportion of participants reported AEs, including transient dizziness and tinnitus during or shortly after pressing; these AEs resolved without medical intervention and did not lead to study withdrawal. In future implementations, instructors should emphasize the precautions for these AEs, such as using alternative fingers and managing the duration of pressure application. The use of acupressure devices, such as acupressure rods, instead of fingers can also help to minimize these adverse events. Additionally, in this study, we acknowledge that AEs may not have been adequately assessed, as they were assessed solely during the second session and at the final follow-up assessment, leaving potential AEs or serious AEs undetected at other follow-up time points. To improve the detection and documentation of AEs in future studies, it would be beneficial to implement a standardized AE checklist tailored to acupuncture-related interventions^58^ and conduct assessments at multiple time points. This approach would enhance the accuracy of AE documentation linked to acupressure.

This RCT had several methodological weaknesses. First, this trial used a partially nested design in which participants received group training, but the sample size was not powered to account for therapist- or group-level clustering, and our primary analysis did not account for such a partially nested design. Second, the lack of a sham control group and the short follow-up period limited our capacity to isolate the specific therapeutic effects of precise acupoint site stimulation and long-term effectiveness. Nonetheless, a pragmatic approach using MHE for comparison allowed us to compare the effects of the SAA training program with those of health education, reflecting real-world help-seeking behavior. However, because participants were not blinded to group allocation and most outcomes (e.g., PHQ-9 and DASS-21 scores, ISI) were self-reported, expectancy and performance biases cannot be excluded and may have inflated the observed differences. However, the blinded researcher-rated HDRS also showed significant results, further suggesting that the improvement was unlikely to be solely attributable to self-reporting bias. To address these limitations, future studies should include a non-acupoint acupressure or sham massage control. This would help to determine whether the benefits of SAA arise specifically from the stimulation of acupoints rather than from tactile contact or the power of expectation. Such a study design would allow for a clearer understanding of the specific effects of acupoint stimulation in accordance with TCM theory. Third, adherence to SAA was assessed using self-reported logbooks. Self-reporting is susceptible to social desirability and recall biases. Similar to other psychological or educational-based interventions (e.g., cognitive behavioral therapy and acceptance and commitment therapy), we could not objectively record participants' practice after the course, but we reminded the participants to report their practice truthfully so we could evaluate the feasibility of implementation. Future studies might consider asking participants to take photos or videos of their practice. This approach could help ensure compliance and allow for better observation of their techniques. Fourth, we did not collect post-intervention follow-up data beyond week 12, so the longer-term benefits remain unknown. The health economic evaluation used a 12-week timeframe, region-specific willingness-to-pay thresholds, and considered solely from the healthcare provider perspective, which limited the generalizability of the cost-effectiveness conclusions to other health systems and longer-term settings. Future research should focus on conducting more comprehensive health economic evaluations with larger sample sizes, longer follow-up periods, and a broader societal perspective. This approach will help to accurately assess the long-term cost-effectiveness and impact of implementing SAA training programs in community settings. Finally, the participants’ depression was assessed on the basis of a questionnaire instead of a clinical diagnosis made by specialists. However, this approach enabled us to evaluate whether the SAA training course can be effectively implemented in communities where clinical diagnosis is not always readily available. In addition, our recruitment through social media and NGOs may be more likely to attract those who are health-conscious or more educated. Consequently, our sample skewed toward female and relatively more educated participants, which may limit the generalizability of our findings. Future studies should employ a more diverse recruitment strategy to ensure a broader representation of the population. Finally, the sample size calculation at the time of study design did not account for clustering within training cohorts or instructors. As a result, the effective sample size in the intervention arm may be reduced, and the study may be underpowered after accounting for intra-class correlation.

In conclusion, this assessor-blind RCT of adults with moderate depressive symptoms provides evidence that the SAA training program is a feasible and potentially effective non-pharmacological intervention for moderate depression. The SAA program conferred a modest additional benefit over MHE, yielding greater improvements in depressive symptoms, insomnia, stress, and quality of life, and enabled more participants to achieve subclinical levels of depression. However, the cost-effectiveness of SAA remains unconfirmed due to the short follow-up period; longer-term follow-up studies are necessary to confirm it.

## Contributors

WFY, BYMY, FYYH, CKHW, HC, YSH, and LMH conceived and designed the study; JYR, and SC acquired the data; WFY, BYMY, and CKHW analyzed and interpreted the data; BYMY and CKHW performed the statistical analysis; WFY and BYMY drafted the manuscript; KFC, DSTC, Z-JZ, SZ, and LL critically revised the important intellectual content of the manuscript. WFY and BYMY have accessed and verified the data. All authors had given the final approval of the manuscript.

## Data sharing statement

All relevant data are provided within the manuscript and its supporting information files.

## Declaration of interests

The researchers have no competing interests.
