# Supplementary material for: Self-administered acupressure training for depression in community-dwelling individuals: a randomized controlled trial and cost-effectiveness analysis
Source: eClinicalMedicine. 2026 Jun 16;96:104023. doi: 10.1016/j.eclinm.2026.104023 (PMC13284438; doi:10.1016/j.eclinm.2026.104023)
Supplement: Supplementary Figure and Tables [file mmc1.docx]

**sFigure S1. Cost-Effectiveness Acceptability Curve**


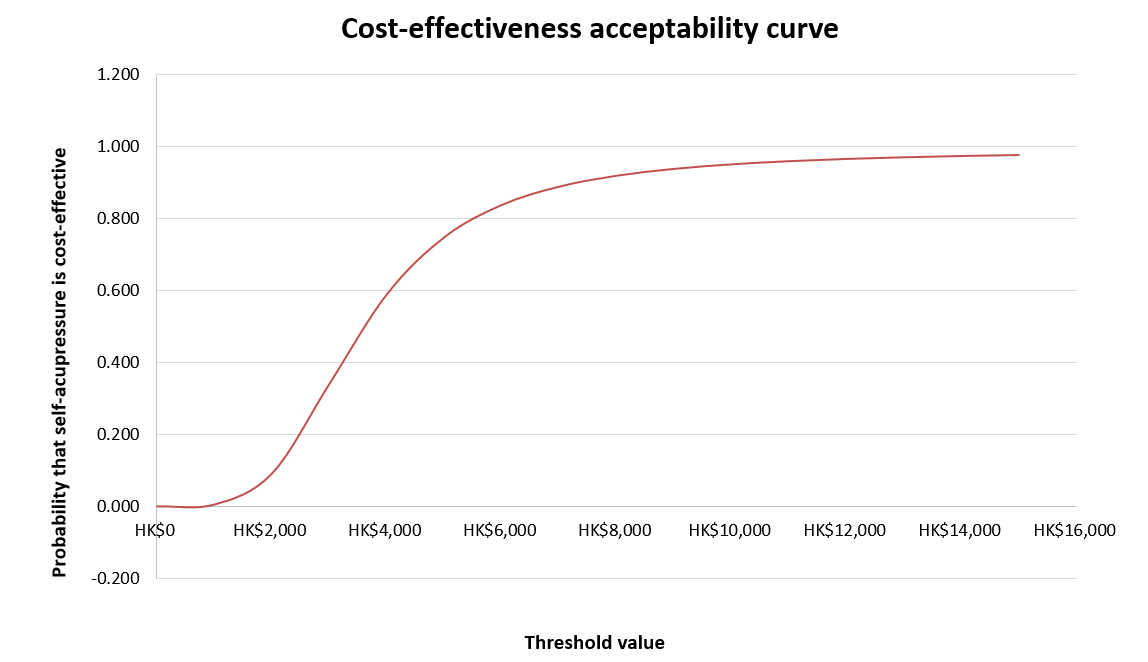


**sTable S1: Participants’ Acupressure Log Summary**

| In the SAA Group (n =125), the number of participants with: | Completed during  Week 1-4,  n (%) | Completed during  Week 5-8,  n (%) | Completed during  Week 9-12,  n (%) | Completed during the 12-week intervention  period |
| --- | --- | --- | --- | --- |
| At least 5 days SAA per week | 101 (80.8%) | 86 (68.8%) | 93 (74.4%) | 71 (56.8%) |
| At least 4 days SAA per week | 109 (87.2%) | 91 (72.8%) | 96 (76.8%) | 77 (61.6%) |
| At least 3 days SAA per week | 115 (92.0%) | 94 (75.2%) | 100 (80.0%) | 82 (65.6%) |
| At least 22 days SAA during the 4-wk period | 108 (86.4%) | 90 (72.0%) | 93 (74.4%) | 76 (60.8%) |
| At least 18 days SAA during the 4-wk period | 114 (91.2%) | 97 (77.6%) | 101 (80.8%) | 86 (68.8%) |
| At least 14 days SAA during the 4-wk period | 118 (94.4%) | 103 (82.4%) | 103 (82.4%) | 94 (75.2%) |

**sTable S2: Depression outcomes across the assessment time points (linear mixed-effects model)**

|  | **SAA group**  **(n = 125)**  **Mean (SE)^a^** | **SAA group**  **change from the baseline**  **Mean (SE)** | **MHE group**  **(n = 125)**  **Mean (SE)^a^** | **MHE group change from the baseline**  **Mean (SE)** | | **Between-group differences in changes from the baseline**  **(95% CI)** | **Effect size (d)^b^** | **P value^c^** |
| --- | --- | --- | --- | --- | --- | --- | --- | --- |
| **PHQ-9 score** | | | | | | | | |
| Baseline | 13.36 (0.36) | - | 13.35 (0.36) | | - | - | - | 0.07^†^ |
| Week 4^d^ | 9.44 (0.37) | -3.93 (0.38) | 10.47 (0.37) | | -2.88 (0.33) | -1.04 (-2.02, -0.06) | 0.27 | 0.04 |
| Week 8^e^ | 8.89 (0.37) | -4.48 (0.43) | 10.13 (0.38) | | -3.22 (0.39) | -1.25 (-2.39, -0.11) | 0.28 | 0.03 |
| Week 12^f^ | 7.89 (0.37) | -5.47 (0.44) | 9.34 (0.38) | | -4.01 (0.42) | -1.46 (-2.65, -0.23) | 0.30 | 0.02 |
| **HDRS score** | | | | | | | | |
| Baseline | 13.38 (0.41) | - | 13.06 (0.41) | | - | - | - | 0.02^†^ |
| Week 4^g^ | 9.23 (0.42) | -4.15(0.42) | 10.56 (0.42) | | -2.50(0.40) | -1.65 (-2.78, -0.52) | 0.36 | 0.004 |
| Week 8^h^ | 9.15 (0.42) | -4.23(0.47) | 10.35 (0.43) | | -2.71(0.45) | -1.53 (-2.81, -0.25) | 0.30 | 0.02 |
| Week 12^i^ | 8.24 (0.42) | -5.14(0.49) | 9.62 (0.43) | | -3.44(0.46) | -1.70 (-3.02, -0.39) | 0.32 | 0.01 |

Abbreviations: CI, confidence interval; HDRS, Hamilton Depression Rating Scale; MHE, mental health education; PHQ-9, Patient Health Questionnaire-9; SAA, self-administered acupressure; SE, standard error.

^a^ Estimated mean and standard error from the linear mixed-effects model; ^b^ Effect size (ES) based on the mean change from the baseline in the treatment group minus the mean change from baseline in the control group, divided by the pooled standard deviation of score change. The ES was categorized as small (ES = 0.2), medium (ES = 0.5), and large (ES = 0.8; Cohen, 1988). A positive sign indicates a highly favorable effect in the intervention group; ^c^ The P value for the interaction between groups and assessment time points in the linear mixed-effects model; ^d^ Due to missing, n = 120 in the SAA group; n = 118 in the MHE group; ^e^ Due to missing, n =118 in the SAA group; n =114 in the MHE group; ^f^ Due to missing, n = 117 in the SAA group; n = 115 in the MHE group; ^g^ Due to missing, n = 120 in the SAA group; n = 116 in the MHE group; ^h^ Due to missing, n = 118 in the SAA group; n = 112 in the MHE group; ^i^ Due to missing, n = 117 in the SAA group; n = 112 in the MHE group;

^†^ The P value for the interaction between groups and all assessment time points in the linear mixed-effects model.

**sTable S3: Sensitivity Analysis of PHQ-9 Score across the Assessment Time Points**

|  | **SAA group**  **(n = 125)**  **Adjusted mean (SE)^a^** | **SAA group**  **change from the baseline**  **Adjusted mean**  **(SE)** | **MHE group**  **(n = 125)**  **Adjusted mean (SE)^a^** | **MHE group change from the baseline**  **Adjusted mean (SE)** | | **Between-group differences in changes from the baseline**  **(95% CI)** | **Effect size (d)^b^** | **P value^c^** |
| --- | --- | --- | --- | --- | --- | --- | --- | --- |
| **PHQ-9 score** | | | | | | | | |
| Baseline | 13.67 (0.40) | - | 13.56 (0.38) | | - | - | - | 0.07^†^ |
| Week 4^d^ | 9.75 (0.40) | -3.91 (0.38) | 10.69 (0.39) | | -2.88 (0.33) | -1.04 (-2.02, -0.06) | 0.26 | 0.04 |
| Week 8^e^ | 9.20 (0.40) | -4.47 (0.43) | 10.35 (0.40) | | -3.21 (0.39) | -1.26 (-2.40, -0.12) | 0.27 | 0.03 |
| Week 12^f^ | 8.20 (0.41) | -5.45 (0.44) | 9.57 (0.40) | | -4.00 (0.42) | -1.47 (-2.65, -0.28) | 0.30 | 0.02 |

Abbreviations: CI, confidence interval; MHE, mental health education; PHQ-9, Patient Health Questionnaire-9; SAA, self-administered acupressure; SE, standard error.

^a^ Estimated mean and standard error from the linear mixed-effects model using current psychiatric disorder and current antidepressant as covariates; ^b^ Effect size (ES) based on the mean change from the baseline in the treatment group minus the mean change from baseline in the control group, divided by the pooled standard deviation of score change. The ES was categorized as small (ES = 0.2), medium (ES = 0.5), and large (ES = 0.8; Cohen, 1988). A positive sign indicates a highly favorable effect in the intervention group; ^c^ The P value for the interaction between groups and assessment time points in the linear mixed-effects model; ^d^ Due to missing, n = 120 in the SAA group; n = 118 in the MHE group; ^e^ Due to missing, n =118 in the SAA group; n =114 in the MHE group; ^f^ Due to missing, n = 117 in the SAA group.

^†^ The P value for the interaction between groups and all assessment time points in the linear mixed-effects model.

**sTable S4: Other study outcomes across the assessment time points (linear mixed-effects model)**

|  | **SAA group**  **(n = 125)**  **Mean (SE)^a^** | **SAA group**  **change from the baseline**  **Mean (SE)** | **MHE group (n = 125)**  **Mean (SE)^a^** | **MHE group**  **change from the baseline**  **Mean (SE)** | **Between-group differences in changes from the baseline**  **(95% CI)** | **Effect size (d)^b^** | **P value^c^** | |
| --- | --- | --- | --- | --- | --- | --- | --- | --- |
| **ISI** | | | | | | | |  |
| Baseline | 15.10 (0.50) | - | 16.00 (0.50) | - | - | - | 0.02^†^ | |
| Week 4^d^ | 10.95 (0.50) | -4.15(0.42) | 13.21 (0.51) | -2.80(0.39) | -1.36 (-2.48, -0.47) | 0.30 | 0.02 | |
| Week 8^e^ | 10.43 (0.50) | -4.67(0.48) | 13.19 (0.51) | -2.82(0.44) | -1.86 (-3.15, -0.58) | 0.36 | 0.005 | |
| Week 12^f^ | 9.37 (0.51) | -5.73(0.50) | 12.07 (0.51) | -3.94(0.46) | -1.80 (-3.14, -0.47) | 0.34 | 0.008 | |
| **DASS-21 Anxiety subscore** | | | | | | | |  |
| Baseline | 15.73 (0.73) | - | 16.67 (0.71) | - | - | - | 0.57^†^ | |
| Week 4^g^ | 11.79 (0.72) | -3.94(0.57) | 13.12 (0.72) | -3.57(0.54) | -0.38 (-1.92, 1.16) | 0.006 | 0.63 | |
| Week 8^h^ | 10.67 (0.72) | -5.05(0.68) | 12.54 (0.73) | -4.14(0.66) | -0.91 (-2.78, 0.96) | 0.12 | 0.34 | |
| Week 12^i^ | 9.48 (0.71) | -6.25(0.72) | 11.85 (0.74) | -4.83(0.72) | -1.43 (-3.44, 0.58) | 0.18 | 0.16 | |
| **DASS-21 Depression subscore** | | | | | | | |  |
| Baseline | 19.95 (0.77) | - | 18.64 (0.77) | - | - | - | 0.02^†^ | |
| Week 4^g^ | 13.87 (0.78) | -6.08(0.63) | 14.98 (0.79) | -3.66(0.61) | -2.42 (-4.14, -0.69) | 0.35 | 0.006 | |
| Week 8^h^ | 13.49 (0.78) | -6.46(0.77) | 14.49 (0.80) | -4.15(0.75) | -2.31 (-4.42, -0.21) | 0.27 | 0.03 | |
| Week 12^i^ | 11.33 (0.79) | -8.62(0.82) | 13.18 (0.80) | -5.47(0.81) | -3.16 (-5.43, -0.89) | 0.43 | 0.007 | |
| **DASS-21 Stress subscore** | | | | | | | |  |
| Baseline | 25.38 (0.78) | - | 24.51 (0.78) | - | - | - | 0.007^†^ | |
| Week 4^g^ | 19.94 (0.79) | -5.44(0.70) | 21.46 (0.80) | -3.08(0.62) | -2.38 (-4.21, -0.55) | 0.32 | 0.01 | |
| Week 8^h^ | 19.53 (0.80) | -5.85(0.82) | 20.92 (0.81) | -3.61(0.73) | -2.25 (-4.42, -0.09) | 0.26 | 0.04 | |
| Week 12^i^ | 16.68 (0.80) | -8.71(0.86) | 19.52 (0.82) | -5.01(0.77) | -3.71 (-5.99, -1.42) | 0.43 | 0.002 | |
| **SF-6D score** | | | | | | | |  |
| Baseline | 0.67 (0.010) | - | 0.67 (0.010) | - | - | - | 0.01^†^ | |
| Week 4^j^ | 0.65 (0.010) | -0.015(0.009) | 0.62 (0.010) | -0.045(0.008) | 0.03 (0.005, 0.054) | 0.32 | 0.02 | |
| Week 8^k^ | 0.68 (0.010) | 0.010(0.011) | 0.65 (0.010) | -0.017(0.009) | 0.03 (-0.002, 0.056) | 0.24 | 0.07 | |
| Week 12^l^ | 0.71 (0.010) | 0.036(0.012) | 0.65 (0.010) | -0.012(0.009) | 0.05 (0.012, 0.078) | 0.41 | 0.002 | |

Abbreviations: CI, confidence interval; DASS-21, Depression Anxiety Stress Scale-21; ISI, Insomnia Severity Index; MHE, mental health education; SAA, self-administered acupressure; SE, standard error; SF-6D, Short Form 6 Dimensions.

^a^ Estimated mean and standard error from the linear mixed-effects model; ^b^ Effect size (ES) based on the mean change from the baseline in the treatment group minus the mean change from baseline in the control group, divided by the pooled standard deviation of score change. The ES was categorized as small (ES = 0.2), medium (ES = 0.5), or large (ES = 0.8; Cohen, 1988). A positive sign indicates a highly favorable effect in the intervention group; ^c^ The P value for the interaction between groups and assessment time points in the linear mixed-effects model; ^d^ Due to missing, n = 120 in the SAA group; n = 115 in the MHE group; ^e^ Due to missing, n =118 in the SAA group; n =112 in the MHE group; ^f^ Due to missing, n = 117 in the SAA group; n = 112 in the MHE group; ^g^ Due to missing, n = 120 in the SAA group; n = 116 in the MHE group; ^h^ Due to missing, n = 118 in the SAA group; n = 112 in the MHE group; ^i^ Due to missing, n = 117 in the SAA group; n = 111 in the MHE group; ^j^ Due to missing, n = 121 in the SAA group; n = 116 in the MHE group; ^k^ Due to missing, n =118 in the SAA group; n =112 in the MHE group; ^l^ Due to missing, n = 117 in the SAA group; n = 111 in the MHE group.

^†^ The P value for the interaction between groups and all assessment time points in the linear mixed-effects model.

| sTable S5. Group Comparison of Participants Who Achieved Clinically Significant Criteria | | | |
| --- | --- | --- | --- |
| Assessment Point | SAA | MHE | *P*-value ^b^ |
|  | *n*/N (%) ^a^ | *n*/N (%) ^a^ |  |
| *PHQ-9 < 5* |  |  |  |
| Week 4 | 17/125 (13.6) | 8/125 (6.4) | 0.06 |
| Week 8 | 23/125 (18.4) | 13/125 (10.4) | 0.07 |
| Week12 | 31/125 (24.8) | 17/125 (13.6) | 0.03 |
| Abbreviations: PHQ-9, Patient Health Questionnaire-9; SAA, Self-administered Acupressure; MHE, Mental Hygiene Education.  ^a^ The number of participants meeting the criterion from the total number of participants analyzed.  ^b^ Group differences were compared with the Chi-square test. | | | |
